# Effects of breast-feeding duration, bottle-feeding duration and non-nutritive sucking habits on the occlusal characteristics of primary dentition

**DOI:** 10.1186/s12887-015-0364-1

**Published:** 2015-04-21

**Authors:** Xiaoxian Chen, Bin Xia, Lihong Ge

**Affiliations:** Department of Pediatric Dentistry, First Dental Center, Peking University School and Hospital of Stomatology, Postal address: Jia No.37 Xishiku Street, Xicheng District, Beijing, 100034 China; Department of Pediatric Dentistry, Peking University School and Hospital of Stomatology, Beijing, 100081 China

**Keywords:** Breast-feeding, Bottle-feeding, Malocclusion, Nursing behavior, Sucking habits, Cross-sectional study

## Abstract

**Background:**

Early transition from breastfeeding and non-nutritive sucking habits may be related to occlusofacial abnormalities as environmental factors. Previous studies have not taken into account the potential for interactions between feeding practice, non-nutritive sucking habits and occlusal traits. This study assessed the effects of breast-feeding duration, bottle-feeding duration and non-nutritive sucking habits on the occlusal characteristics of primary dentition in 3–6-year-old children in Peking city.

**Methods:**

This cross sectional study was conducted via an examination of the occlusal characteristics of 734 children combined with a questionnaire completed by their parents/guardians. The examination was performed by a single, previously calibrated examiner and the following variables were evaluated: presence or absence of deep overbite, open bite, anterior crossbite, posterior crossbite, deep overjet, terminal plane relationship of the second primary molar, primary canine relationship, crowding and spacing. Univariate analysis and multiple logistic regressions were applied to analyze the associations.

**Results:**

It was found that a short duration of breast-feeding (never or ≤6 months) was directly associated with posterior cross bite (OR = 3.13; 95% CI = 1.11–8.82; P = 0.031) and no maxillary space (OR = 1.63; 95% CI = 1.23–2.98; P = 0.038). In children breast-fed for ≤6 months, the probability of developing pacifier-sucking habits was 4 times that for those breast-fed for >6 months (OR = 4.21; 95% CI = 1.85–9.60; P = 0.0002). Children who were bottle-fed for over 18 months had a 1.45-fold higher risk of nonmesial step occlusion and a 1.43-fold higher risk of a class II canine relationship compared with those who were bottle-fed for up to 18 months. Non-nutritive sucking habits were also found to affect occlusion: A prolonged digit-sucking habit increased the probability of an anterior open bite, while a pacifier-sucking habit associated with excessive overjet and absence of lower arch developmental space.

**Conclusion:**

Breastfeeding duration was shown to be associated with the prevalence of posterior crossbite, no maxillary space in the deciduous dentition and development of a pacifier-sucking habit. Children who had a digit-sucking habit were more likely to develop an open bite.

## Background

Craniofacial growth and development are affected by functional stimuli such as sucking, chewing, swallowing and breathing [1]. Nutritive sucking, which includes breast-feeding and bottle-feeding and non-nutritive sucking (NNS), which includes pacifier and digit sucking, have been associated with growth and development of the maxillomandibular complex. Breast-feeding has been cited as one of the environmental factors responsible for correct development of dentofacial structures [2]. Recently, the biomechanics of milk extraction during breast-feeding have been investigated in detail. One group of researchers used an objective and dynamic analysis of ultrasound (US) videos acquired during breast-feeding to show that this complicated procedure requires coupling between the periodic motions of the infant’s jaws, the undulation of the tongue, and the breast-milk ejection reflex [3]. Another group found that extraction of milk during breast-feeding involved development of time-varying sub-atmospheric pressures within the infant’s oral cavity, and that vacuum pressures as low as −145 mmHg may be generated [4]. Conversely, absence or short duration of breast-feeding results in the child doing fewer oral exercises; this leads to underdevelopment of the muscles, incorrect posture of the lip and tongue, and the acquisition of harmful oral habits, all of which may be associated with dental malocclusions [2].

An absence or short duration of breast-feeding results in a longer duration of bottle-feeding. The sucking mechanism used during bottle-feeding is markedly different from that used during breast-feeding [1,5,6]. Compared with breast-feeding, bottle-feeding requires less forceful muscle action and thus, does not facilitate mandibular development to the same degree. This difference could potentially predispose those children who undergo prolonged bottle-feeding to malocclusion or other distinctive occlusion characteristics [1]. However, more evidence is needed to support this association.

The empirical literature regarding the link between feeding practice and occlusal problems is far from unanimous in its conclusions. Some studies have reported that breast-feeding is a protective factor against malocclusion: Labbok and Hendershot have suggested that increased duration of breast-feeding is associated with a decline in the proportion of children with malocclusion [7], and Adamiak has linked it with a reduced need for orthodontic treatment [8]. In non-breast-fed children, the occurrence of an anterior open bite has been found to be significantly increased, demonstrating the beneficial influence of breast-feeding on dental occlusion [2]. However, other studies have indicated no relationship between the duration of breast-feeding in the first year of life and any dental arch or occlusal parameters [9]. Several reports have suggested that bottle-feeding may be responsible for the development of non-nutritive sucking habits and that these may be responsible for some forms of malocclusion [5,10,11]. Previous studies may not have taken account of confounding factors such as interactions between feeding practice, non-nutritive sucking habits and occlusal traits. Currently, the association between bottle-feeding and the incidence of occlusal alterations in the sagittal plane remains up for debate.

In Peking, the proportion of children receiving at least four months of exclusive breast-feeding is reported to be as low as 39.2%, with an average weaning time of only 7.66 months [12]. Chinese studies on the feeding practice in childhood and their effects on occlusal characteristics, particularly in Peking city, are lacking. Therefore, the aim of this study was to assess, using multivariate analysis, the possible relationships between breastfeeding duration, bottle-feeding duration, non-nutritive sucking habits and dental arch characteristics in children aged 3–6 years old with primary dentition and attending state preschools in Peking, China.

## Methods

### Subjects and sample

The data for this cross-sectional study were obtained from annual oral health examinations in two daycare centers, located at Tsinghua University and Peking University, from April to May 2014. The sample size was calculated using the sample size formula and was based on the data from a previous prevalence study. In this study by Takuro, the prevalence of anterior crossbite was 5.7% in the children who were bottle-feeding at 24 months of age [13]. A desired accuracy of 10% and a significance level of 5% were adopted. We determined a minimum sample of 516 children. However, to compensate for possible non-responses, losses and failures to meet inclusion criteria, the study population was increased by 30% to 671 children.

### Questionnaire

This study was approved by the Research Ethics Committee at Peking University School and Hospital of Stomatology (Protocol No. PKUSSIRB-201414045). Written informed consent was obtained from parents or guardians of each subject.

Based on questionnaires designed by Charchut et al. [14] and answered by the parents/guardians, a retrospective investigation was made concerning the age and sex of the children, the parents’ schooling level, the feeding method (breast and/or bottle) used (including duration of use) for each 6-month period making up the first 36 months of the child’s life and finally, the child’s non-nutritive sucking habits during these time periods and at present. Feeding frequency was graded according to the following categories: method frequently used, used moderately, infrequently used, not used. Since a child might have received nutrition via both methods, the child was considered to be bottle-fed when the bottle score was higher than the breast score. A child was considered to exhibit non-nutritive sucking habits if he/she had been sucking an object (usually a digit or a pacifier) not related to feeding for more than the first year of life [5].

### Inclusion criteria

The inclusion criteria were as follows: completely filled out questionnaire with no items missed, complete primary dentition without missing teeth, absence of extensive caries affecting the mesio-distal and occluso-gingival dimensions, absence of stainless steel crowns, absence of dental morphological anomalies, number and structure, absence of erupted or erupting permanent teeth, absence of trauma to the craniofacial complex, no history of orthodontic treatment and cooperation during the examination. The aim of all of the above criteria was to exclude factors that would compromise occlusal relationships or interfere with the examination results.

### Occlusal examination

The occlusal examinations were performed by a single examiner, a calibrated dentist who was blind to the questionnaire data. An intra-examiner reliability test was performed by examining 20 children at two different times, 2 weeks apart, and the Kappa value was calculated to be 85%. The clinical examinations were performed in the daycare center, using a mouth mirror and probe, under a suitably directed portable light source. The children lay down in a comfortable position, while the examiner was seated at 12 o’clock. The occlusal relationships were examined by direct visual inspection of the teeth at maximum mouth opening and then at maximum intercuspation. The primary second molar relationships and primary canine relationships were recorded while the patients’ primary teeth were closed at maximum intercuspation.

The following arch characteristics were recorded by a single examiner throughout the study using published definitions [15,16]:

1. Overbite was graded according to coverage of the mandibular incisor by the most protruded fully erupted maxillary incisor and recorded as <1/2, or ≥1/2.
2. Anterior open bite was recorded when one or more of the maxillary incisors occluded lingual to the mandibular incisors.
3. Overjet or horizontal overlap was measured from the palatal surface of the mesial corner of the most protruded fully erupted maxillary incisor to the labial surface of the corresponding mandibular incisor. The degree of overjet was recorded in millimeters. In this study, an overjet of greater than 4 mm was considered an increased overjet.
4. Posterior crossbite was recorded when one or more of the maxillary primary canines or molars occluded lingual to the buccal cusps of the opposing mandibular teeth.
5. The terminal relationship of the deciduous second molar were classified into three categories: flush terminal, where the distal surfaces of the upper and lower second primary molars are in the same vertical plane in a centric occlusion; distal step, where the distal surfaces of the lower primary second molar are in a posterior relationship to the distal surface of the upper second molars in centric occlusion; mesial step, the distal surfaces of the lower primary second molar are in an anterior relationship to the distal surface of the upper second molars in centric occlusion. Flush terminal and distal step relationships were combined as nonmesial step relationships.
6. Canine relationship was classified into three categories: class I, the tip of the maxillary primary canine tooth is in the same vertical plane as the distal surface of the mandibular primary canine; class II, the tip of the maxillary primary canine tooth is mesial to the distal surface of the mandibular primary canine; class III, the tip of the maxillary primary canine is distal to the distal surface of the mandibular primary canine.
7. Spacing: If primate space or developmental space was present, spacing was noted.
8. Crowding: When there are one or more teeth with disturbance of position or rotation, crowding was noted.

In determining the occlusal relationships of the primary second molar and primary canine, the occurrence of a similar occlusion on both sides was taken as a criterion. In the determination of the primary second molar relationship, if one side had a flush terminal plane while the other side had a distal or mesial step, which was noted as a distal or mesial terminal plane. In determining the canine relationship, if there was a class II or class III relationship on one side and class I on the other side, that was considered a class II or class III relationship. Children with a mesial step on one side and a distal step on the other were excluded from the study.

### Data analysis

A descriptive analysis was carried out, in which the differences in the distribution of the study covariates in the dependent variable categories were assessed. The chi-square test was used to analyze the associations between breast-feeding duration, bottle-feeding duration, non-nutritive sucking and occlusal characteristics, at significance levels of P < 0.05.

To measure the strength of the association and the relative chances of developing a particular occlusion characteristic, the odds ratio (OR) was calculated in a multivariate logistic regression analysis. Arch characteristics were the dependent variables, including anterior open bite, anterior crossbite, posterior crossbite, no maxillary space, mesial step, class II canine relationship, excessive overjet and space. Duration of breast-feeding was the main independent variable and was classified as either ‘never breast-fed/breast-fed until the age of six months’ or ‘breast-fed for more than six months’. The covariates were duration of bottle-feeding (≦18 months or >18 months), age (3–4, 4–5 or 5–6 years), sex, digit sucking (‘never had this habit/had this habit but stopped before the age of one’ or ‘had this habit for longer than one year’) and pacifier sucking (classification as for digit-sucking). A confidence interval of 95% was used as the criterion for statistical inference.

## Results

Originally, we recruited 847 Chinese children from 3–6 years of age with complete primary dentition. Of these, 113 were excluded because their questionnaires were not completely/correctly filled in. Most questionnaires (93.2%) were completed by the parents. The remaining (6.8%) questionnaires were completed by the grandfather or grandmother.

The demographic and descriptive results for the subjects are presented in Table 1. Our sample included 734 children: 398 males and 336 females with a mean age of 4.48 ± 0.84. In most cases (73.4%), at least one of the parents had postgraduate qualifications. The incidence of NNS was 23.2%. The proportion of children who were breast-fed for 1–6 months was 27.1%, never breastfed was 13.8%. The proportion of children who were bottle-fed for over 18 months was 58.4%, bottle-fed for 6–18 months was 41.6%. None of children in the present study was bottle-fed for 0–6 months. In the first 6 months, 14.2% of infants were exclusively bottle-fed, 22.9% were exclusively breast-fed while 62.9% were mixed-fed.

**Table 1:** **Characteristics of the study group**

**variable(n = 734)**	**Number**	**%**
Age (years)	4.48 ± 0.84	
3-4	354	48.2
4-5	164	22.4
5-6	216	29.4
Children sex		
Male	398	54.2
Female	336	45.8
Level of education		
Bachelor degree or below	195	26.6
Masters	199	27.1
Doctorate	340	46.3
Non-nutritive sucking habits		
Pacifier sucking	30	4.1
12-36 months	27	3.7
>36 months	3	0.4
Digit sucking	140	19.1
12-36 months	53	7.2
>36 months	87	11.9
Duration of breast-feeding		
Never breastfed	101	13.8
1-6 months	199	27.1
>6 months	434	59.1
Duration of bottle-feeding		
0-6 months	0	0
6–18 months	305	41.6
>18 months	429	58.4
Percentage of each feeding practice in first 6 months		
exclusive breastfeeding	168	22.9
exclusive bottle-feeding	104	14.2
mixed feeding	462	62.9

An increased percentage of posterior crossbite and no maxillary space (no upper arch space) were found in children who were breast-fed for less than 6 months (Table 2). No association was found between the duration of breast-feeding and the other examined occlusal characteristics. Analysis of the multivariate regression revealed that breast-feeding for no longer than 6 months seems to be the most important factor influencing posterior crossbite (OR = 3.13; 95% CI = 1.11–8.82; P = 0.031) and no maxillary space (OR = 1.63; 95% CI = 1.23–2.98; P = 0.038).

**Table 2:** **Logistic regression analysis of the association between feeding duration and occlusal characteristics**

**Characteristics**		**Breast-feeding duration**					**Bottle-feeding duration**				
		**0-6 months**	**>6 months**				**6-18 months**	**>18 months**			
		**n (%)**	**n (%)**	**OR**	**95% CI**	**P**	**n (%)**	**n (%)**	**OR**	**95% CI**	**P**
Posterior crossbite	yes	11(3.7)	6(1.4)				9(3.0)	8(1.9)			
	no	289(96.3)	428(98.6)	3.13	1.11-8.82	0.031*	296(97.0)	421(98.1)	2.03	0.73-5.66	0.173
No maxillary space	yes	110(36.7)	114(26.3)				83(27.2)	130(30.3)			
	no	190(63.3)	320(73.7)	1.63	1.23-2.98	0.038*	222(72.8)	299(69.7)	1.13	0.80-1.58	0.485
Nonmesial step	yes	108(36.0)	157(36.2)				181(59.3)	288(67.1)			
	no	192(64.0)	277(63.8)	0.97	0.71-1.33	0.862	124(40.7)	141(32.9)	1.45	1.06-2.1	0.018*
Class IIcanine relationship	yes	92(30.7)	135(31.1)				81(26.6)	146(34.0)			
	no	208(69.3)	299(68.9)	0.94	0.68-1.31	0.713	224(73.4)	283(66.0)	1.43	1.03-2.0	0.034*
Anterior open bite	yes	3(1.0)	4(0.9)				3(1.0)	4(0.9)			
	no	297(99.0)	430(99.1)	0.87	0.18-4.26	0.861	302(99.0)	425(99.1)	0.61	0.12-3.18	0.559
Anterior crossbite	yes	25(8.3)	37(8.5)				30(9.8)	32(7.5)			
	no	289(96.3)	428(98.6)	0.93	0.54-1.61	0.792	275(90.2)	397(92.5)	0.67	(0.39-1.16)	0.152

Multivariate logistic regression analysis was used to the calculate odds ratio (OR) of breastfeeding duration and bottle-feeding duration developing a particular occlusion characteristic.

*Comparisons are significantly different, P < 0.05.

Bottle-feeding for longer than 18 months was associated with an increased prevalence of a nonmesial terminal plane (OR = 1.45; 95% CI = 1.06–2.0; P = 0.018) and of a class II canine relationship (OR = 1.43; 95% CI = 1.03–2.0; P = 0.034), compared with children who were bottle-fed for between 6 and 18 months (Table 2). No association was found between duration of bottle-feeding and any other occlusal characteristics.

The effects of the feeding method on non-nutritive sucking habits were quite different (Table 3). Our data indicated that children who were breast-fed for less than 6 months showed a higher probability of pacifier sucking (OR = 4.21; 95% CI = 1.85–9.60; P < 0.001). However, breast-feeding did not increase the probability of having a digit sucking habit (OR = 0.89; 95% CI = 0.61–1.30; P = 0.54). Bottle-feeding for longer than 18 months did not increase the prevalence of pacifier-sucking habit or digit-sucking habit.

**Table 3:** **Feeding practice and non-nutritive sucking habits**

**Non-nutritive habit**		**Breast-feeding**					**Bottle-feeding**				
		**0-6 months n(%)**	**>6 months n(%)**	**OR**	**95% CI**		**6-18 months n(%)**	**>18 months n(%)**	**OR**	**95% CI**	**P**
						**P**					
Pacifier sucking	yes	22(73.3)	8(26.7)				14(46.7)	16(53.3)			
	no	278(39.5)	426(60.5)	4.21	1.85-9.60	0.0002*	291(41.3)	413(58.7)	1.24	0.59-2.58	0.562
Digit sucking	yes	54(38.6)	86(61.4)				56(40.0)	84(60.0)			
	no	246(41.4)	348(58.6)	0.89	0.61-1.30	0.538	249(41.9)	345(58.1)	0.92	0.64-1.34	0.679

Multivariate logistic regression analysis was used to the calculate odds ratio (OR) of breastfeeding duration and bottle-feeding duration developing pacifier or digit sucking habit.

*Comparisons are significantly different, P < 0.05.

The prevalence of posterior crossbite and no maxillary space did not increase significantly in children with pacifier sucking or digit-sucking habits, when compared to children without these habits (Table 4). Similarly, there was no significant effect on the prevalence of a nonmesial terminal plane and a class II canine relationship. This suggests that NNS does not act in synergy with feeding practice to promote the development of the positive occlusal/arch characteristics mentioned above. A pacifier-sucking habit that lasted beyond one year of age was associated with excessive overjet (P = 0.01), absence of lower arch developmental space (P = 0.03). While a digit sucking habit that lasted beyond one year of age was associated with anterior open bite (P < 0.001).

**Table 4:** **Non-nutritive sucking habits and occlusal characteristics**

**Characteristics**	**Pacifier sucking**		**Digit sucking**	
	**Chi-square test**	**P**	**Chi-square test**	**P**
Posterior crossbite	0.7416	0.3891	1.2050	0.2723
No maxillary space	0.0146	0.9038	0.9173	0.3382
Mesia step	0.1041	0.7470	0.0114	0.9151
Class IIcanine relationship	0.2657	0.6062	0.0036	0.9519
Overjet≧4 mm	6.6105	0.0101*	0.0305	0.8613
Absence of lower arch developmental space	4.3492	0.0370*	2.3139	0.1282
Anterior open bite	0.3012	0.5832	20.3337	0.0000*
Anterior crossbite	0.2477	0.6187	0.1574	0.6916

The chi-square test was used to analyze the associations between non-nutritive sucking and occlusal characteristics.

*Comparisons are significantly different, P < 0.05.

## Discussion

The literature provides no single criterion for malocclusion of primary dentition; therefore most occlusal characteristics were assessed in determining the relationship between malocclusion and feeding patterns. Our findings indicated that failure to breast-feed or breast-feeding for only a short period was associated with a higher prevalence of posterior crossbite and no maxillary space in the primary dentition. Sucking the breast places great demands on the perioral musculature. The constant repetitive effort promotes the correct development of these muscles, increasing their tone and ensuring that correct oral function is established. As a result, the duration of natural breast-feeding has a positive effect on the mobility of orofacial structures [17]. Early weaning may lead to an insufficient perioral muscular activity, which may cause negative consequences to swallowing, breathing and speaking, as well as malocclusion [18]. Warren et al. found that breast-feeding facilitates normal palate development and attenuates the formation of a deep, high-arched palate [10]. Viggiano et al. and Karjalainen et al. have reported that breast-feeding was a protective factor against development of posterior crossbite in the deciduous dentition [5,19]. Similarly, Kobayashi et al. reported that prolonged exclusive breast-feeding can strongly reduce the prevalence of posterior crossbite, and children who were breast-fed for more than 12 months had a 20-fold lower risk of posterior crossbite compared with children who were never breast-fed. Additionally, their risk was 5-fold lower than those who were breast-fed for between 6 and 12 months [20]. This evidence corroborates the findings of the present study. Although the absolute incidence of posterior crossbite was extremely low, its harmful effects on masticatory chewing cycle pattern and normal growth and development of the orofacial system were noticeable [21]. We suggest that early weaning may interfere with the normal development of alveolar ridges and the hard palate, and hence result in posterior crossbite, a lack of space or crowding in the upper arch.

The parents were very highly educated in our study and the rate of exclusive breast-feeding of children at 6 months of age was 22.9%. The corresponding rate in rural western China was 11.6 % [22], which was lower than our study. Scott et al. reviewed literature identifying factors associated with the initiation and duration of breastfeeding among Western women and found there was a strong and consistent association with demographic factors such as maternal age and level of education, there was a less consistent association with factors such as marital and socioeconomic status. [23]. However, Agboado et al. found there were no significant associations between breastfeeding cessation and marital status, mode of delivery, timing of breastfeeding initiation and socio-economic deprivation in Lancashire, UK [24]. These different finding highlights the fact that breastfeeding is multifactorial in nature and future programs aimed at promoting breastfeeding must take this into consideration.

Our study found a significant association between non-mesial step and bottle-feeding for over 18 months, compared to children who were bottle-fed for less than 18 months. A similar association was also found between the class II canine relationships and bottle-feeding. It is known that the terminal relationship of primary second molars seems to be the most important factor that could determine or influence the future relationship between the permanent molars and the subsequent development of occlusion. As Moyers and Waiuright reported, non-mesial step combined with a class II canine relationship increases the probability of developing Angle class II malocclusion in mixed dentition and permanent dentition [25]. Therefore, in many cases, early orthodontic intervention will be needed. Similarly, Nahás-Scocate et al. found that the older the child when bottle-feeding ceased (3–4 years old) and the shorter the breastfeeding duration (<3 months), the greater the chances of the child presenting distal step [26]. There are several theoretical mechanisms by which bottle-feeding might contribute to the development of malocclusion: (1) less muscle activity is necessary to extract milk from a bottle, resulting in decreased development of muscles involved in sucking, which may act as a functional matrix for inadequate mandibular growth; (2) The tongue acts only to control the milk outlet during bottle-feeding and bottle-fed children have an increased prevalence of abnormal swallowing patterns or tongue thrusting habits [27]; (3) over 60% of the children who were predominantly bottle-fed presented mouth breathing or mixed breathing, which may compromise occlusion [28]. Although Narbutyte et al. [29] found that the literature contains insufficient evidence to connect bottle-feeding with the development of skeletal malocclusions, the results of our study clearly showed that bottle-feeding may be related to abnormal maxillomandibular relationship via its inadequate provision of muscular stimulation. Nevertheless, the absolute OR value was low. The reason may be that the effects of bottle-feeding on occlusion were difficult to assess when bottle-feeding was concurrent with breast-feeding. In this study, 14.2% of infants were exclusively bottle-fed in the first 6 months while 62.9% were mixed-fed. This would weaken the effect of bottle-feeding on any specific type of malocclusion. Nevertheless, there was a weak trend associating bottle-feeding for over 18 months with inadequate mandibular growth.

The incidences of pacifier sucking and digit sucking in the present study were 4.1% and 19.1%, respectively. Previous studies on non-nutritive sucking habits have shown a variation in prevalence between 20% and 87% at age 36 months [20,25,30]. Pacifier and digit sucking incidences have been reported to be 18.2% and 61.5% in Australia [31], 70.3% and 10% in Sweden, and 50% and 19% in Norway [32]. Most previous studies have reported much higher incidences of NNS compared with our study. This difference suggests that the incidence of NNS varies from one population to another and may be related to local culture and customs. The present study showed that 40.9% children were weaned early and this increased the prevalence of pacifier sucking by up to 4.1 fold, but it did not increase the prevalence of digit sucking. Theories that endeavor to explain this effect suggest that children who are naturally breast-fed satisfy their sucking needs along with their psychological and affective requirements through close, intimate contact with the mother during breast-feeding and thus have less need to suck a pacifier, digit or other object [33]. Conversely, non-nutritive sucking habits are developed when children satisfy their instinctive sucking urge by sucking their thumbs or by prolonged use of pacifiers. Nobuya et al. also reported that pacifier sucking was more prevalent in children with a short breast-feeding duration than in those with a normal feeding duration [34]. Luz et al. examined 249 children with mixed dentition and found statistically significant associations between short breast-feeding duration (<6 months) and non-nutritive sucking habits, and between non-nutritive sucking habits and class II malocclusions [35]. Montaldo et al. reported that children who underwent bottle- or complementary feeding showed a higher risk of acquiring non-nutritive sucking habits after the first year of life, and that this was associated with a greater risk of crossbite, open bite and class II molar relationships [36]. Surprisingly no correlation was found between bottle-feeding duration and pacifier sucking or between bottle-feeding duration and digit sucking. This can be explained by the fact that Chinese parents will usually select a milk bottle or other way to calm their infants rather than a pacifier.

Different sucking habits are known to affect occlusion in different ways, and the disturbances found in the present study bear similarities to other findings elsewhere. In this study, a significant correlation was seen between pacifier sucking and excessive overjet as well as absence of lower arch developmental space. Pacifier sucking appeared to have an effect on the lower labial segment. Similar trends were reported by Warren et al., who found an association between prolonged pacifier sucking and a shorter intercanine width in the mandibular arch [10]. Aznar et al. found that a pacifier-sucking habit led to a significant reduction in the intercanine and intermolar mean width in the maxillary arch [37]. Melink et al. compared 30 children with a unilateral posterior crossbite and 30 children without a crossbite and found that the duration of pacifier habits were associated with posterior crossbites at the age of 4 or 5 years because of low tongue posture in the mouth [38]. These inconsistent findings can be explained by the force and frequency of muscle action involved in different pacifier sucking pattern or duration, which result in different effect on the development of the dental arches. About half of the children in the present study were 3–4-years old and sucking a pacifier was rare in those beyond 3 years of age, so they may be too young to present an anterior open bite. Moreover, as was mentioned above, Chinese parents seldom use a pacifier to placate their infants, so the number with the habit was not large enough to show the most serious and representative effects.

The finding that pacifier- and digit-sucking habits have different effects on occlusal traits has also been reported previously [10,39,40]. The present study found that digit sucking resulted in an increased prevalence of anterior crossbite and that it had a significant effect on the upper labial segment. Franco also found that for the first 3–4 years of life, the detrimental effect on occlusion is largely confined to the anterior segment [41]. The effect on development of dentofacial structures depends on the duration and frequency of the habit, the intensity of the sucking, the relationship of the dental arches and the direction and nature of the force exerted by the digit [42]. Generally, it is agreed that prolonged digit sucking is associated with increased overjet, greater maxillary arch depth, and greater prevalence of anterior open bite [37,40,41]. The present study pointed to the association between digit sucking and anterior open bite. This might be because a digit-sucking habit is difficult to give up and 62% of the children with the digit-sucking habit maintained it up to 3 years of age; this makes it more likely to impact occlusion and lead to vertical disturbances. Our study also showed that non-nutritive sucking habits were not associated with the prevalence of posterior crossbite and no maxillary space, nor did we find any effects on mesial step and class II canine relationship. We suggest that feeding practice may play a primary role in the development of posterior crossbite and no maxillary space. The evidence presented in this study suggests that children should be predominant breast-fed or exclusive breast-fed for no less than 6 months where possible and that parent should be more aware of the potentially deleterious effects of non-nutritive sucking habits on oral development. From another direction, our finding fitted in with the WHO recommendation of breastfeeding exclusively for 6 months.

However, there are some intrinsic limitations of cross section study and it was hard to take into consideration of the effect of heredity and other oral detrimental habits on malocclusion. The recall bias of the people who filled the questionnaire still existed, which might affect the accuracy of the duration of feeding practice. A future study with a larger sample size and isolating single variables is needed for a thorough understanding of the relationship between sucking and malocclusions.

## Conclusions

The present study highlights the importance of taking into account multiple interactions between feeding practice, non-nutritive sucking habits and occlusal characteristics. The results suggest that even in the absence of non-nutritive sucking habits, failure to breast-feed for a sufficient length of time may negatively affect maxillary arch growth and may lead to malocclusion in the form of a posterior crossbite. Another negative consequence may be a prolonged pacifier-sucking habit, as we found that the probability of this increased 4-fold in children who were breast-fed for less than 6 months. In addition, we have found that increased bottle-feeding duration may contribute to inadequate mandibular development, and that non-nutritive sucking habits can be a dominant and deleterious factor in the development of occlusofacial problems.
